# “Intensity-Response” Effects of Electroacupuncture on Gastric Motility and Its Underlying Peripheral Neural Mechanism

**DOI:** 10.1155/2013/535742

**Published:** 2013-07-01

**Authors:** Yang-Shuai Su, Wei He, Chi Wang, Hong Shi, Yu-Feng Zhao, Juan-Juan Xin, Xiao-Yu Wang, Hong-Yan Shang, Ling Hu, Xiang-Hong Jing, Bing Zhu

**Affiliations:** ^1^Institute of Acupuncture and Moxibustion, China Academy of Chinese Medical Sciences, 16 Nanxiaojie, Dongzhimennei, Beijing 100700, China; ^2^Department of Gastroenterology, Peking University First Hospital, 6 Xishiku Street, Beijing 100034, China; ^3^Institute of Basic and Clinic Medicine, China Academy of Chinese Medical Sciences, 16 Nanxiaojie, Dongzhimennei, Beijing 100700, China

## Abstract

The aim of this study was to explore the “intensity-response” relationship between EAS and the effect of gastric motility of rats and its underlying peripheral neural mechanism by employing ASIC3 knockout (ASIC3−/−), TRPV1 knockout (TRPV1−/−), and C57BL/6 mice. For adult male Sprague-Dawley (*n* = 18) rats, the intensities of EAS were 0.5, 1, 3, 5, 7, and 9 mA, respectively. For mice (*n* = 8 in each group), only 1 mA was used, by which C fiber of the mice can be activated. Gastric antrum motility was measured by intrapyloric balloon. Gastric motility was facilitated by EAS at ST36 and inhibited by EAS at CV12. The half maximal facilitation intensity of EAS at ST36 was 2.1–2.3 mA, and the half maximal inhibitory intensity of EAS at CV12 was 2.8 mA. In comparison with C57BL/6 mice, the facilitatory effect of ST36 and inhibitive effect of CV12 in ASIC3−/− mice decreased, but the difference was not statistically significant (*P* > 0.05). However, these effects in TRPV1−/− mice decreased significantly (*P* < 0.001). The results indicated that there existed an “intensity-response” relationship between EAS and the effect of gastric motility. TRPV1 receptor was involved in the regulation of gastric motility of EAS.

## 1. Introduction

Acupuncture therapy, as a traditional Chinese medicinal treatment, has been widely used in clinical practice in oriental countries. And it has been more accepted by practitioners and patients worldwide after its therapeutic effects for the treatments of postoperative dental pain, nausea, and vomiting have been confirmed by NIH in 1997 [1]. Electroacupuncture (EA) is a modification of conventional manual acupuncture to stimulate acupoints with electrical current. It appears to induce more consistently reproducible effects in both clinical and animal researches than manual acupuncture [2].

During the last decades, a large number of studies have been performed to investigate the effects of acupuncture on gastrointestinal secretion, motility, and gastric myoelectrical activity [3–5]. Some regular responses of gastrointestinal tract induced by acustimulation have been observed in various studies. In animal models, acupuncture at hindlimb has been reported to accelerate delayed gastric emptying [6], restore impaired gastric accommodation in vagotomized dogs [7], and relax the gastric fundus in rats [8] via the parasympathetic pathway, whereas application of acupuncture at the abdomen was more likely to inhibit gastrointestinal motility [9, 10] via the sympathetic pathway. Most studies have mainly focused on whether acupuncture treatment is effective for restoring gastrointestinal disorders. However, few were performed to explore the “intensity-response” relationship between electroacupuncture stimulation (EAS) and the effect. In the present paper, according to the threshold of activating peripheral III (A*δ*) and IV (C) primary afferent fibers [11], EAS with different intensities was introduced to reveal the “intensity-response” effects between EAS and the effect of gastric motility.

Previous studies showed that A*β* and A*δ* mechanical receptors, as well as C-polymodal receptors, played important roles in the acupuncture stimulation perception [12–14]. But what afferent fibers mediate the regulatory effect of EAS on the internal organs was ignored. Acid sensing ion channel 3 (ASIC3) is a member of the DEG/ENaC family which is known to mediate mechanical responsiveness [15] and located mainly in A*β* primary afferent fibers innervating the skin and muscle [12, 16]. Transient receptor potential vanilloid (TRPV1) belongs to TRPV subfamily, which is expressed in sensory A*δ* and C fibers. It can be activated by capsaicin, noxious heat, low PH, and voltage and closely related to noxious physical detection [17–19]. In our previous study, these two knockout mice have been utilized to observe the effect of EAS on mechanical and thermal pain thresholds, which showed that both EA and thermal stimulation of the right ST36 can raise mechanical and thermal pain thresholds in TRPV1−/− and C57BL/6 mice, but stimulation should be more stronger in TRPV1−/− mice [20].

In the present study, both ASIC3 knockout (ASIC3−/−) mice and TRPV1 knockout (TRPV1−/−) mice were employed to establish dysfunction of A*β* and A*δ*/C afferent fiber mice models, respectively, in order to investigate the roles of A*β* and A*δ*/C fibers in the EAS-modulated gastric motility.

## 2. Materials and Methods

### 2.1. Animal Preparation

Male Sprague-Dawley (SD) rats (*n* = 18), weighing 250–300 g, were purchased from Institute of Animal, Academy of Chinese Medical Sciences. Male ASIC3−/− mice (*n* = 8), TRPV1−/− mice (*n* = 8), and C57BL/6J mice (*n* = 8), weighing 25–30 g, were purchased from Jackson Lab (USA) and bred at the China Academy of Chinese Medical Science Animal Care Facility. The animals were housed under a 12 h light/dark with free access to food and water. All animals were treated according to the Guide for Use and Care of Medical Laboratory Animals from Ministry of Public Health of People's Republic of China.

### 2.2. Gastric Motility Recording

The animals were fasted overnight with free access to water. For anesthesia, 10% urethane (1.0–1.2 g/kg, via intraperitoneal route) was administered. About 1 h after the urethane administration, the animals were under deep anesthesia, and the trachea was cannulated but not immobilized to keep respiratory tract unobstructed. A catheter was inserted into one of the jugular veins for infusion. A small longitudinal incision was made in the duodenum about 1 cm from the pylorus. A small balloon made of flexible condom rubber was inserted via incision of the duodenum into the pyloric area of rat and kept in position by tying the connecting catheter to the duodenum. And another catheter (inner diameter of 1 mm) was also inserted into the same hole by incision in order to drain digestive juices secreted from stomach. The balloon was filled with about 0.2–0.3 mL warm water to keep pressures at about 100 mmH_2_O. For the operation of the mice, a smaller balloon filled with 0.05–0.08 mL warm water was inserted into the pyloric area to keep the pressures at about 100 mmH_2_O.

Pressure in the balloon was measured by a transducer through a thin polyethylene tube (1.5 mm in outer diameter) and then input into a polygraph amplifier (NeuroLog, NL900D). The signal was captured online and analyzed offline using a data acquisition system (Power-Lab/4s, AD Instruments) and Chart 5.2 software. Demifasting gastric motor activity was recorded as a control for at least 30 min before any stimulation. The gastric motility induced by EAS was compared with the background activity in terms of average amplitude (the average difference between the cyclic maxima and minima in the selected cycles), integral (returns the integral of the selection, calculated as the sum of the data points multiplied by the sample interval), and frequency (per minute) of gastric contraction waves. Systemic blood pressure and heart rate were continuously monitored by using of BIOPAC data acquisition system (MP150, USA), and rectal temperature kept constantly around 37°C by a feedback-controlled heating blanket (DC, USA).

Gastric motility during and after EAS was compared with background activity. If the change rates of gastric motility during or after EAS were 15–20% of the basal activity, the response was then considered to have an excitatory or inhibitory effect. The first EA stimulus was applied when gastric motility wave maintained stable, usually at about 30 minutes after the surgical procedure. Different intensities of EAS, including 0.5 mA (<T_A*δ*_), 1 mA (<T_A*δ*_), 3 mA (>T_A*δ*_, <T_C_), 5 mA (>T_C_), 7 mA (>T_C_), and 9 mA (>T_C_), were applied at ST36 or CV12 in an ascending order. The latter stimulus can only be applied when the gastric motility recovered to control state. The background gastric activity and gastric activity during and after EAS were recorded continuously, 60 s for each session. 

### 2.3. Electroacupuncture Stimulation (EAS) of CV12 and ST36

Rats were randomly divided into ST36 group (*n* = 9) and CV12 group (*n* = 9). A needle (0.3 mm in diameter) was inserted into the skin and its underlying muscles at acupoints Zhongwan (CV12) and Zusanli (ST36) on the body. CV12 was located at center of abdomen, in middle line of the body. ST36 was located bilaterally at the anterior tibia muscles near the knees. EAS was performed at unilateral ST36 or CV12 for 60 s. A pair of noninsulated needle electrodes inserted into the skin of the acupoints with 0.3 cm distance. The needles were connected to an electronic stimulator (SEN-7103, Nihon Kohden) with the parameters as follows: duration: 1 ms, pulse frequency: 15 Hz. For rats, the current intensities were 0.5, 1, 3, 5, 7, and 9 mA, respectively. For mice, only 1 mA EAS was administrated.

### 2.4. Statistical Analysis 

Changes in the average amplitude and integral were calculated according to (the value during EAS-the value pre EAS)/the value pre EAS × 100%. The data obtained before and after treatment in the same group or different group was compared statistically by a paired *t*-test or unpaired *t*-test. *P* < 0.05 was considered as a statistical significance. All data are expressed as mean ± SE.

The data was fitted with (1), where *C* is set to be 500, *a* is set to be 50, and *b* is set to be 30:

(1) $$\begin{array}{c} Y=\frac{C}{\left(1+\exp\left(a-b\right)*X\right)}. \end{array}$$

## 3. Results

### 3.1. Gastric Motility under Resting Condition

The gastric motility of the rats and mice was detected by recording the intragastric pressure. When the intrapyloric balloon pressure was increased to about 80–200 mmH_2_O, the rhythmic waves of contractions in pyloric area were observed. With regard to gastric motor characteristics, both the changes of intragastric pressure and rhythmic contraction were noteworthy. Generally, the intragastric pressure represents the index of gastric tone motility, and rhythmic contraction represents gastric peristalsis induced by circular muscle contractions, similar to slow wave of gastric motor activity. The pressure was maintained at about 100 mmH_2_O as baseline by expanding the volume of the balloon with warm water, rhythmic contractions occurred at a rate of four to six per minute, and these rhythmically gastric contractions were recorded in both the rats and mice.

### 3.2. Facilitatory Effect of Gastric Motility Induced by ST36 and Its Intensities Response Effects of the Rats

**Figure 1:**
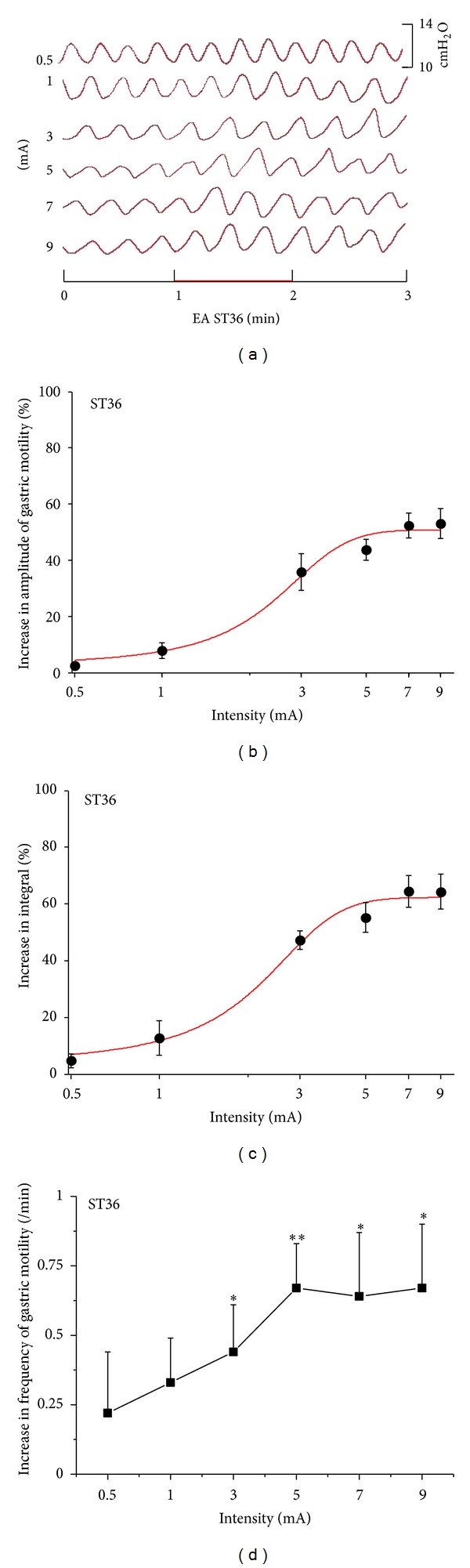
Gastric motility in response to EAS at ST36 with different intensities in rats. (a) Representative examples of the alterations of gastric contraction wave induced by different intensities of EAS at ST36. (b), (c), and (d) displayed the facilitatory effects of EAS at ST36 on the amplitude, integral, and frequency of gastric motility, respectively (*n* = 9; *P < 0.05, **P < 0.01, versus background activities).

EAS at ST36 induced facilitatory effects which were related to the intensities.  Figure 1(a) showed typical responses of gastric motility following EAS with various intensities for 60 s. Figures 1(b) and 1(c) summarized the responses obtained from all 9 tested rats. It should be noted that when the stimulation was less than 1 mA, there was no significant response of gastric motility (amplitude changes: 0.5 mA: 2.4 ± 1.1%, 1 mA: 4.7 ± 2.4%, *P* > 0.05) (integral changes: 0.5 mA: 7.8 ± 2.8%, 1 mA: 12.7 ± 5.8%, *P* > 0.05). However, 3 mA, 5 mA, 7 mA, and 9 mA EAS at ST36 elicited a significant enhancement on the amplitude and integral of gastric contraction compared with the background activities (amplitude changes: 3 mA: 37.9 ± 5.8%, 5 mA: 43.7 ± 3.7%, 7 mA: 52.3 ± 4.4%, 9 mA: 53.1 ± 5.4%, *P* < 0.01, *P* < 0.001) (integral changes: 3 mA: 47.2 ± 3.2%, 5 mA: 55.2 ± 5.3%, 7 mA: 64.9 ± 5.6%, 9 mA: 64.3 ± 6.2%, *P* < 0.001). The facilitation of EAS at ST36 appeared from a low intensity with an EC_50_ value of approximately 2.3 mA for amplitude (Figure 1(b)) and 2.1 mA for integral (Figure 1(c)), which means that EAS with 2.1–2.3 mA can obtain 50% of the maximum facilitatory effect. For the intensity of EAS above 5 mA, the response efficiency did not increase correspondingly as intensities increasing, which indicated that the effects may hit a “plateau region” when the stimulating intensity reached to a certain level.

Figure 1(d) illustrated the impact of EAS at ST36 on the frequency of gastric motility. Intensity of EAS lower than 1 mA failed to produce any significant response (frequency changes: 0.5 mA: 0.22 ± 0.22/min, 1 mA: 0.33 ± 0.16/min, *P* > 0.05), while 3 mA, 5 mA, 7 mA, and 9 mA EAS at ST36 induced significant enhancement on the frequencies of gastric motility compared with the background activities (frequency changes: 3 mA: 0.44 ± 0.17/min, 5 mA: 0.67 ± 0.16/min, 7 mA: 0.64 ± 0.23/min, 9 mA: 0.67 ± 0.23/min, *P* < 0.05, *P* < 0.01). The maximal facilitatory response of the frequency appeared as the intensities reached to 5 mA.

### 3.3. Inhibitory Effect of Gastric Motility Induced by CV12 and Its Intensities Response Effect of the Rats

**Figure 2:**
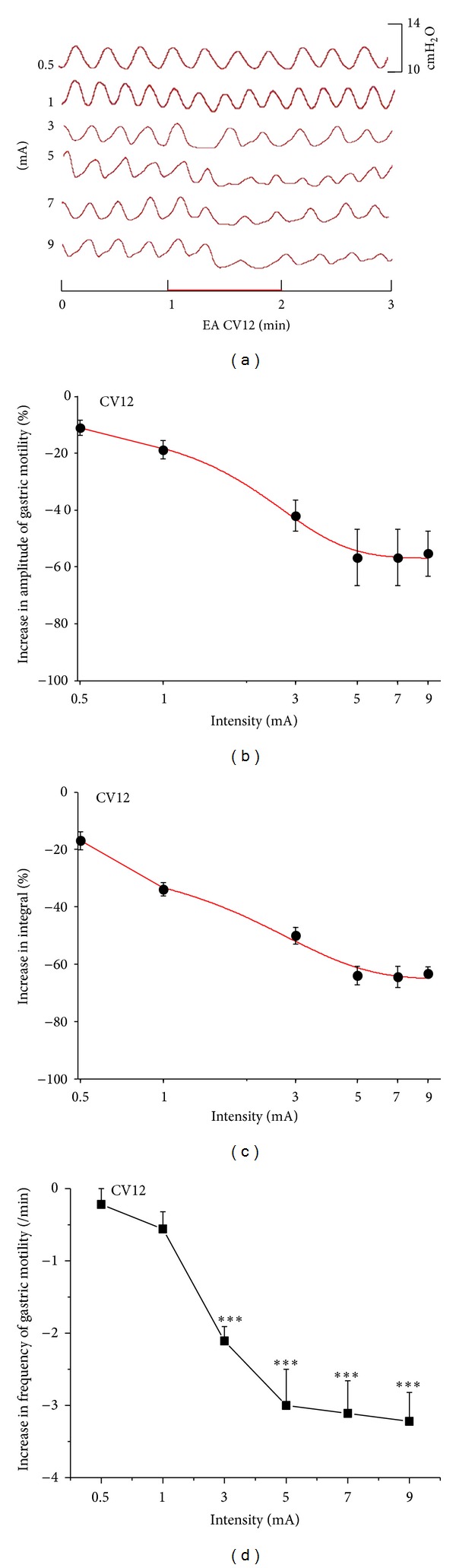
Gastric motility in response to EAS at CV12 with different intensities in rats. (a) Representative examples of the alterations of gastric contraction wave induced by different intensities of EAS at CV12. (b), (c), and (d) displayed the inhibitory effects of EAS at CV12 on the amplitude, integral, and frequency of gastric motility, respectively (*n* = 9; ***P < 0.001 versus background activities).

EAS at CV12 induced inhibitory effects which were also related to the intensities.  Figure 2(a) showed typical responses of gastric motility following EAS with different intensities for 60 s, and Figures 2(b) and 2(c) summarized the responses obtained from all 9 tested rats. EAS with all the intensities at CV12 induced significant inhibition effects on the amplitudes and integrals of gastric contraction (amplitude: 0.5 mA: −11.1 ± 2.7%, *P* < 0.05; 1 mA: −18.8 ± 3.2%, 3 mA: −42.0 ± 5.5%, 5 mA: −56.7 ± 10%, 7 mA: −56.3 ± 10%, and 9 mA: −57.3 ± 7.2%, *P* < 0.01) (integral: 0.5 mA: −17.0 ± 3.2%, *P* < 0.01; 1 mA: −34.0 ± 2.3%, 3 mA: −50.1 ± 3%, 5 mA: −64.4 ± 3.2%, 7 mA: −64.0 ± 3.7%, and 9 mA: −63.4 ± 2.5%, *P* < 0.001). The inhibition of EAS at CV12 appeared from a low intensity (0.5 mA), with IC_50_ value of approximately 2.8 mA for both amplitude and integral (Figure 2(b)). This means that EAS with 2.8 mA can obtain 50% of the maximum inhibitory effect. When the intensity reached to 5 mA, the response efficiency did not increase correspondingly. The “plateau region” also appeared in the CV12 which induced the inhibitory effects.

Figure 2(d) displayed the impact of EAS on the frequency of gastric motility by CV12. Intensities of EAS lower than 1 mA had no significant influence on the frequencies (frequency changes: 0.5 mA: −0.22 ± 0.22/min, and 1 mA: −0.54 ± 0.24/min, *P* > 0.05). But 3 mA, 5 mA, 7 mA, and 9 mA EAS at CV12 induced a significant inhibition on the frequency of gastric motility compared with the background activities (frequency changes: 3 mA: −2.11 ± 0.22/min, 5 mA: −3.0 ± 0.5/min, 7 mA: −3.11 ± 0.45/min, and 9 mA: −3.22 ± 0.42/min, *P* < 0.01). The inhibitory response of the frequency was prone to be maximal when the intensity reached to 5 mA.

### 3.4. Facilitatory and Inhibitory Effects of EAS on Gastric Motility Require ASIC3 and TRPV1 Receptors

**Figure 3:**
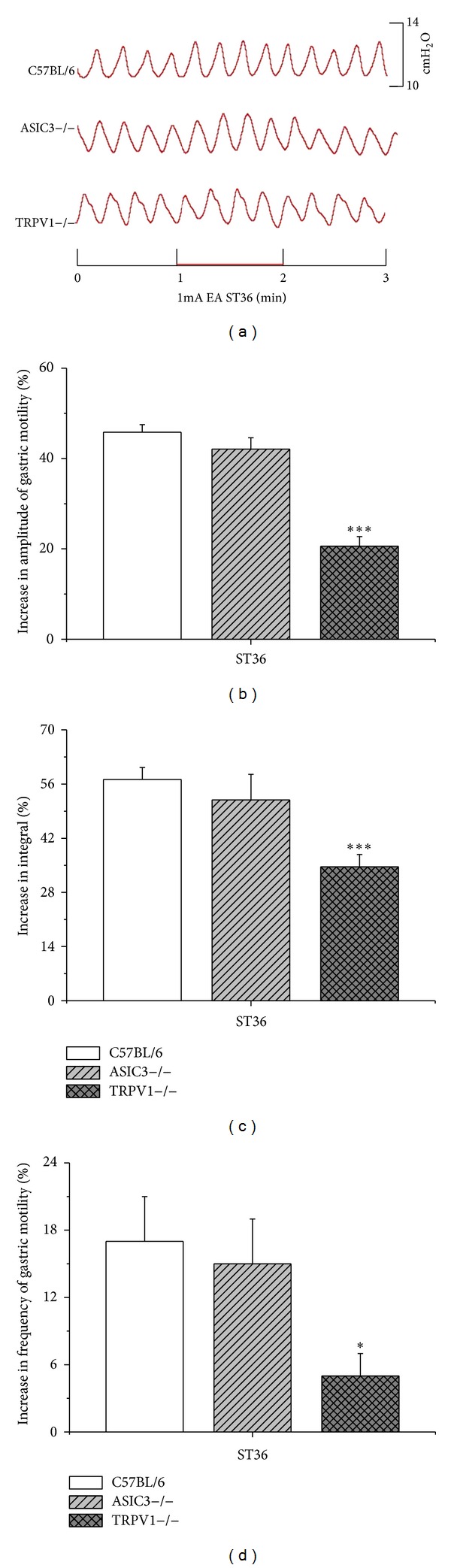
Gastric motility in response to 1 mA EAS at ST36 in three groups of mice. (a) Representative examples of the alterations of gastric contraction wave induced by 1 mA EAS at ST36. (b), (c), and (d) displayed the comparison of the facilitatory effects of 1 mA EAS at ST36 on the amplitude, integral, and frequency of gastric motility, respectively, among three groups of mice (C57BL/6, *n* = 8; ASIC3−/−, *n* = 8; TRPV1−/−, *n* = 8; *P < 0.05, ***P < 0.001 versus C57BL/6).

**Figure 4:**
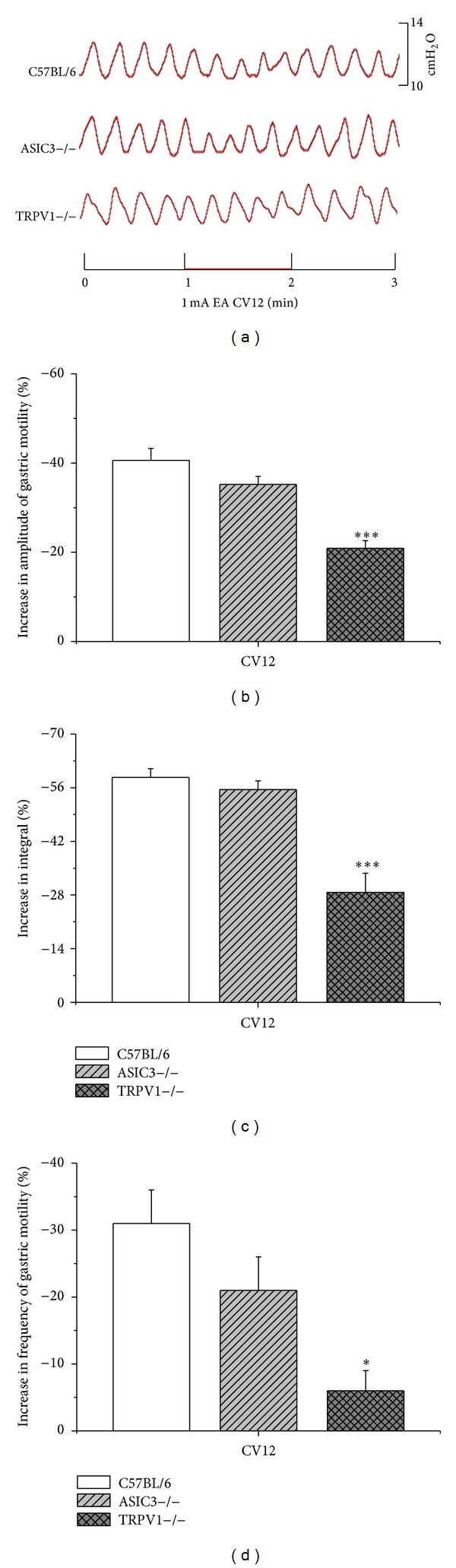
Gastric motility in response to 1 mA EAS at CV12 in three groups of mice. (a) showed representative examples of the alterations of gastric contraction wave induced by 1 mA EAS at CV12. (b), (c), and (d) displayed comparison of the inhibitory effects of 1 mA EA at CV12 on the amplitude, integral, and frequency of gastric motility, respectively, among three groups of mice (C57BL/6, *n* = 8; ASIC3−/−, *n* = 8; TRPV1−/−, *n* = 8; *P < 0.05, ***P < 0.001 versus C57BL/6).

The previous data showed that there existed a possibility of “intensity-response” relationship between stimulation and effects of gastric motility. We speculated that the EAS with intensities of activation A*δ* and C fiber played important roles for modulating gastric motility. According to the threshold of C fiber of mice [21], 1 mA was administrated. EAS with 1 mA at ST36 induced facilitatory effects of gastric motility, and the amplitude as well as integral increased by 45.8 ± 1.7% and 57.2 ± 3.1%, respectively, in C57BL/6 mice. Notably, the facilitatory effects partly diminished in ASIC3 and TRPV1 knockout mice (Figures 3 and 4). The facilitatory effects reduced a little in ASIC3−/− mice but markedly in TRPV1−/− mice (amplitude: 20.6 ± 2.1%; integral: 34.6 ± 3.2%, *P* < 0.001, Figures 3(b) and 3(c)) compared with that in C57BL/6 mice so did the inhibitory effects by CV12 in ASIC3−/− and TRPV1−/− mice (*P* < 0.001, Figures 4(b) and 4(c)). The frequency increased by 17.5 ± 3.8% in C57BL/6 mice via 1 mA EAS at ST36. The facilitatory effects on frequency slightly reduced in ASIC3−/− mice but significantly in TRPV1−/− mice (frequency: 5 ± 2.1%, *P* < 0.05, Figure 3(d)) so did the inhibitory effects by CV12 in ASIC3−/− and TRPV1−/− mice (*P* < 0.05, Figure 4(d)). Taken together, these observations provided direct evidence for the role of TRPV1, rather than ASIC3, in EAS-mediated facilitatory and inhibitory effects on gastric motility.

## 4. Discussion

In the present study, we investigated the “intensity-response” relationship between EAS and the effect of gastric motility in rats. And we firstly observed which afferent fibers were involved in the effect of EAS on gastric motility by using of knockout mice. Our findings strongly indicated the existence of  “intensity-response” effects of EAS on gastric motility. EAS at ST36 induced facilitatory effects which were related to the intensities. After data fitting, the EC_50_ (the half maximal facilitation intensity) of EAS at ST36, was 2.1–2.3 mA, which was near the threshold of A*δ* fiber. EAS at CV12 displayed inhibitory effects which were also related to the intensities. The IC_50_ (the half maximal inhibitory intensity) of EAS at CV12, was about 2.8 mA, which was also near the threshold of A*δ* fiber. These data suggested that the activation of A*δ* fiber was important for EAS-modulated gastric motility. Further study in ASIC3 and TRPV1 knockout mice showed that both ASIC3 and TRPV1 receptors were involved in the effects of EAS on gastric motility, but there was a quantity difference in the changes of gastric motility between ASIC3 and TRPV1 knockout mice. TRPV1 played a more important role in the effects of EAS. 

Based on another experiment in our research group, 1 mA was strong enough to activate the C primary afferent fiber in mice. The different gastric responses induced by 1 mA EAS between ST36 and CV12 were mainly caused by diverse somatoautonomic reflexes; that is, the facilitatory effect of EA at ST36 was mediated via the parasympathetic pathway, whereas the inhibitory effect of EA at abdomen was reasoned to be attributed to the sympathetic pathway. The involvement of the opioidergic pathway has also been frequently reported [18, 22]. EA was more likely to activate various afferent fibers of rats including groups II-III [19], groups III-IV [23], or groups II–IV [24]. Recent study showed that the subepidermal nerve fibers showed the colocalization of TRPV1 with peripherine, a marker for the C and A*β* fibers. Relationship between TRPV1 and effects of acupuncture was further investigated recently. Our previous study suggested an involvement of TRPV1 receptors in acupuncture analgesia [20]. Wang et al. showed that EA at ST36 and ST37 reduces zymosan-induced colorectal hypersensitivity through regulating TRPV1 expression [25]. Moreover, the expression of TRPV1 in subepidermal nerve fibers was significantly increased by EAS at BL40, which indicated that TRPV1 may play a role in local effect of the EA [26]. According to the result of this study, the modulatory effects of EAS at both ST36 and CV12 were barely changed in ASIC3−/− mice compared with C57BL/6 mice. However, the potency of stimulating these two acupoints decreased significantly in TRPV1−/− mice. These results suggested that A*δ* and C fiber were more critical than A*β* fiber in the effects of EA-modulated gastric motility. In another somatovisceral reflex study, Noguchi et al. revealed that to decrease duodenal motilities, EAS to the abdomen needed to be strong enough to excite group IV (C) fibers of intercostal nerves. To increase motilities, EAS to the hindpaw needs to be strong enough to excite the higher threshold group III (A*δ*) fibers of tibial nerves. Their results also indicated the critical roles of A*δ* and C primary afferent fibers in effective regulation of EAS on visceral organ, which were quite similar to our results [25].

It is generally believed that acupuncture at different acupoints produces different effects, and the site-specific inhibitory or facilitatory effects of acupuncture on gastric motility had already been proposed [22, 27, 28]. In the present study, we found that EAS with different intensities at ST36 induced facilitatory responses of gastric motility, whereas EAS at CV12 produced an inhibitory impact on gastric motility. The consistent results have been reported in previous studies [17, 29]. The facilitatory effects of EAS at ST36, as well as inhibition effects of EAS at CV12, ranged from 20% to 60% approximately. The effects reached saturation when the intensity got to a certain level. It was also manifested that EAS had a relative narrow band control for the gastric motility and EAS modulation was a kind of self-limiting and self-regulation to promote the regulation of homeostasis of the body, which demonstrated that EAS modulation is a safe therapy. 

## 5. Conclusion

There existed “intensity-response” relationship between stimulation and effects on gastric motility. TRPV1 receptor was involved in the regulation process of EAS. It is necessary to activate A*δ* fiber to get remarkable modulatory effects, and these effects tended to maximization when C fiber was activated.
